# Gut microbiota alterations are associated with functional outcomes in patients of acute ischemic stroke with non-alcoholic fatty liver disease

**DOI:** 10.3389/fnins.2023.1327499

**Published:** 2023-12-20

**Authors:** Gaojie Yu, Qionglei Chen, Jiaxin Chen, Xiaolan Liao, Huijia Xie, Yiting Zhao, Jiaming Liu, Jing Sun, Songfang Chen

**Affiliations:** ^1^Department of Geriatrics, The Second Affiliated Hospital and Yuying Children’s Hospital of Wenzhou Medical University, Wenzhou, Zhejiang, China; ^2^Department of Preventive Medicine, School of Public Health and Management, Wenzhou Medical University, Wenzhou, Zhejiang, China; ^3^Department of Neurology, The Second Affiliated Hospital and Yuying Children’s Hospital of Wenzhou Medical University, Wenzhou, Zhejiang, China

**Keywords:** ischemic stroke, non-alcoholic fatty liver disease, gut microbiota, biomarker, adverse prognosis, prediction

## Abstract

**Introduction:**

Patients with acute ischemic stroke (AIS) with non-alcoholic fatty liver disease (NAFLD) frequently have poor prognosis. Many evidences suggested that the changes in gut microbiota may play an important role in the occurrence and development of AIS patients with NAFLD. The purpose of this study was to explore microbial characteristics in patients of AIS with NAFLD, and the correlation between gut microbiota and functional outcomes.

**Methods:**

The patients of AIS were recruited and divided into NAFLD group and non-NAFLD group. The stool samples and clinical information were collected. 16 s rRNA sequencing was used to analyze the characteristics of gut microbiota. The patients of AIS with NAFLD were followed-up to evaluate the functional outcomes of disease. The adverse outcomes were determined by modified Rankin scale (mRS) scores at 3 months after stroke. The diagnostic performance of microbial marker in predicting adverse outcomes was assessed by recipient operating characteristic (ROC) curves.

**Results:**

Our results showed that the composition of gut microbiota between non-NAFLD group and NAFLD group were different. The characteristic bacteria in the patients of AIS with NAFLD was that the relative abundance of *Dorea*, *Dialister*, *Intestinibacter* and *Flavonifractor* were decreased, while the relative abundance of *Enorma* was increased. Moreover, the characteristic microbiota was correlated with many clinical parameters, such as mRS scores, mean arterial pressure and fasting blood glucose level. In addition, ROC models based on the characteristic microbiota or the combination of characteristic microbiota with independent risk factors could distinguish functional dependence patients and functional independence patients in AIS with NAFLD (area under curve is 0.765 and 0.882 respectively).

**Conclusion:**

These findings revealed the microbial characteristics in patients of AIS with NAFLD, and further demonstrated the predictive capability of characteristic microbiota for adverse outcomes in patients of AIS with NAFLD.

## 1 Introduction

Acute ischemic stroke (AIS) is a severe and common cerebrovascular disease in humans, which is the main cause of death and disability (Kim et al., 2022). AIS patients frequently have concomitant non-alcoholic fatty liver disease (NAFLD), which significantly worsens their prognosis (Hu et al., 2018; Duell et al., 2022). Recent studies have revealed that NAFLD plays a pivotal role in the occurrence and development of stroke (Bisaccia et al., 2023). The estimated prevalence of NAFLD in general people is approximately 20–30% (Estrada et al., 2019), while the rate rises to 40–50% in patients with initial stroke (Canillas et al., 2022). The incidence and prevalence of NAFLD were not only positively related to stroke onset (Chen H. et al., 2022), but also associated with stroke severity and progression (Li H. et al., 2018). The patients with NAFLD were prone to develop stroke and frequently accompanied by systemic inflammation (Gao and Tsukamoto, 2016), which contributed to the neurological deficits in stroke mice models (Li et al., 2020). Furthermore, a prior study observed that AIS patients with NAFLD showed severe neurological impairment and worse outcomes compared to non-NAFLD patients (Abdeldyem et al., 2017). Nowadays, the pathophysiological mechanisms of NAFLD affecting the clinical outcomes of AIS patients remain elusive and the early identification of adverse outcome in AIS patients with NAFLD is an urgent issue that needs to be explored.

Growing evidence supported the significant alterations of the diversity and component of gut microbiota in stroke patients compared to healthy subjects (Xiang et al., 2020). Gut microbiota have been revealed to be an important contributing factor in the development and prognosis of stroke in mice models (Benakis et al., 2016; Singh et al., 2016). Our previous studies revealed an increase in bacteria secreting pro-inflammatory factors and a decrease in bacteria producing short chain fatty acids (SCFAs) in post-stroke cognitive impairment (PSCI) patients compared to the non-PSCI patients (Ling et al., 2020). In addition, our another studies revealed that characteristic gut microbiota were tightly associated with clinical outcomes in AIS patients with hyperlipidemia (Chen J. et al., 2022), and post-stroke depression in AIS patients (Yao et al., 2023). Moreover, the ability of gut microbiota to distinguish disease status had been conformed in many neuropsychiatric diseases. A classifier based on the abundance of gut microbiota and the clinical parameters identified Parkinson’s disease patients with pretty sensitivity and specificity (Scheperjans et al., 2015). Notably, regulating of gut microbiota dysbiosis by fecal microbiota transplantation could improve the symptoms of Parkinson’s disease in mice (Sun et al., 2018). The crosstalk between the gut and brain was observed between the gut and liver, when the imbalance of gut microbiota elicited the liver to be in a diseased state and as a part of bidirectional interaction the diseased liver in turn shaped the composition and function of gut microbiota (Albillos et al., 2020; Tilg et al., 2022). Abnormal gut microbiota had been observed in NAFLD patients and was associated with NAFLD severity (Boursier et al., 2016). Decreased abundance of bacteria producing butyrate in NAFLD could result in increased gut permeability, endotoxemia, and systemic inflammation (Chen and Vitetta, 2020), with increased risk for brain impairment (Wang et al., 2022). The alteration of gut microbiota and relevant metabolites induced by high-fat in animal models could elicit neurotoxic injuries resulting in metabolic and functional disruption of brain (Higarza et al., 2019). It was reported that specific microbiota compositions influenced by western diet administration could be explored as an indicator for NAFLD progression (Zhuge et al., 2022). Fecal microbiota transplantation was found to decrease the fat accumulation in the liver to attenuate NAFLD by improving the gut microbiota dysbiosis in a randomized clinical trial (Xue et al., 2022). Although NAFLD might be involved in the occurrence and development of AIS through gut microbiota, the key bacteria of AIS patients with NAFLD and their relationships with AIS outcomes have not been explored.

In this study, we explored the characteristic gut microbiota in AIS patients with NAFLD, further evaluated the relationship between characteristic microbiota and clinical parameters, and determined the gut microbial signature to predict adverse outcomes in patients of AIS with NAFLD.

## 2 Methods

### 2.1 Patients recruitment

Patients with first-episode AIS were recruited in the Second Affiliated Hospital of Wenzhou Medical University from September 2020 to July 2021. A total of 184 AIS patients were recruited according to the following inclusion criteria: AIS patients admitted within 3 days of symptom onset diagnosed by trained neurologists based on imaging and clinical evidence (Mendelson and Prabhakaran, 2021); aged 35–85 years; without history of drinking; had conducted abdominal ultrasonography at admission; without the use of probiotics or antibiotics in the last 3 months; without special dietary habits such as dieting or vegetarianism. Finally, 149 recruited patients remained according to the following exclusion criteria: severe gastrointestinal diseases, such as inflammatory bowel disease, gastrointestinal hemorrhage, etc. and history of gastrointestinal surgery; serious organs dysfunction; history of malignancy; tuberculosis; hepatitis virus or alcohol or drug or autoimmunity associated liver disease; failed to follow-up. Then AIS patients were divided into non-NAFLD group and NAFLD group. The diagnosis of NAFLD was based on ultrasonographic findings of liver and the absence of a history of excessive alcohol consumption. The ultrasonographic manifestation of NAFLD is enhanced near-field echo and attenuated far-field echo in the liver region (Adams et al., 2005). This study was approved by the Medical Ethics Committee of the Second Affiliated Hospital of Wenzhou Medical University.

### 2.2 Parameters and samples collection

All recruited patients underwent baseline surveys and follow-up. Surveys were conducted by medical professionals in a question-and-answer format at admission, in order to collect information about socio-demographic characteristics, e.g., age, gender, marital status, educational level, medical histories, e.g., hypertension, diabetes, hyperlipidemia, risk factors, e.g., body mass index (BMI), blood pressure, smoking, drinking, and neuropsychological assessments, e.g., Hamilton Anxiety Rating Scale (HAMA), Hamilton Depression Rating Scale (HAMD), Pittsburgh Sleep Quality Index (PSQI), Mini-Mental State Examination (MMSE), National Institute of Health Stroke Scale (NIHSS). The fasting blood samples were collected within 24 h of patient admission to detect laboratory parameters such as C-reactive protein (CRP), hypersensitive C-reactive protein (Hs-CRP), fasting blood glucose (FBG), glycosylated hemoglobin (HbA1c), alanine transaminase (ALT), aspartate transaminase (AST), uric acid, homocysteine (Hcy), triglycerides (TG), total cholesterol (TC), high-density lipoprotein (HDL), low-density lipoprotein (LDL). The fresh fecal samples from first defecation were collected after admission, and each fecal sample (200 mg) was placed in a 2 mL sterile centrifuge tube. Recruited patients were followed-up by Modified Rankin Scale (mRS) scores to evaluate the functional outcomes at 3 months post-stroke. The mRS score ≥ 2 were defined as functional dependence (FD), and < 1 were defined as functional independence (FI). Drinking was defined as having drunk for more than 1 year and had an average alcohol consumption ≥50 mL/d.

### 2.3 Fecal samples disposition and gut microbiota analysis

Fecal samples were collected, then stored at−80°C. The procedures of fecal samples disposition as follows: DNA extraction using the E.Z.N.A.® soil DNA Kit (Omega Bio-tek, Norcross, GA, U.S.) according to manufacturer’s protocols, PCR amplification with primers 338F9 (ACTCCTACGGGAGGCAGCAG) and 806R (GGACTACHVGGGTWTCTAAT), sequencing of purified amplicons on the Illumina MiSeq platform, quality-controlled, clustering, and annotation of the raw data. More details had been illustrated in our previous studies (Chen J. et al., 2022). The abundance and diversity of gut microbiota in non-NAFLD and NAFLD groups were analyzed on ASV level. The index Ace and Shannon were used to reflect alpha diversity of gut microbiota. Beta diversity was analyzed using principal coordinate analysis (PCoA). The Wilcoxon rank sum test was used to examine characteristic microbiota in NAFLD group. Linear discriminant analysis effect size (LEfSe) was used to estimate the effect of relative abundance of each species on inter-group differences and linear discriminant analysis (LDA) > 2 as threshold for screening characteristic bacteria. Correlation between clinical parameters and gut microbiota was analyzed using Spearman’s rank correlation.

### 2.4 Statistical analysis

Statistical analyses were performed by SPSS software (version 27.0). The quantitative data with normal distribution were presented as mean ± standard deviation, while quantitative data with skewed distribution were denoted by media with interquartile range. Qualitative data were expressed as number with percentage. Student’s *t*-test or Mann–Whitney U-test was used to compare continuous variables, and Chi-square test was performed for categorical variables assessment. Multivariate logistic regression model was built to explore the influence of clinical parameters on FD at 3 months post-stroke after adjusting age, hypertension, diabetes, and hyperlipidemia. Relative risks were expressed as odd ratios (OR) with 95% confidence intervals (CI). Recipient operating characteristic (ROC) curves were used to assess the predictive power of gut microbiota reflected by the area under the curve (AUC). *p* values <0.05 were considered significant.

## 3 Results

### 3.1 The comparison of clinical parameters between NAFLD and non-NAFLD groups

Baseline characteristics of patients were shown in Table 1. AIS patients with NAFLD (*n* = 64, 43.0%) tended to be younger (median 65 years versus 67 years) and were more likely to be women (53.1% versus 48.2%). The prevalence of hypertension and diabetes in NAFLD group were significantly higher than non-NAFLD group (*p* = 0.013, *p* = 0.010, respectively). Moreover, the level of TG was significantly higher compared to non-NAFLD group (*p* < 0.001) and HDL was significantly lower compared to non-NAFLD group (*p* = 0.009). In addition, the BMI, Hs-CRP, FBG, HbA1c, ALT, uric acid, and percentage of FD were also significantly different between the two groups (all *p* < 0.05). The marital status, educational level, neuropsychological assessments at admission were no significant differences between the two groups. As shown in Table 2, the multivariate logistic regression analysis revealed the existence of NAFLD (OR = 2.359, *p* = 0.043), female (OR = 2.910, *p* = 0.014) and elevated NIHSS score (OR = 1.461, *p* < 0.001) were independent risk factors of FD at follow-up.

**Table 1:** The comparison of clinical parameters between NAFLD and non-NAFLD groups.

Parameter	NAFLD (*n* = 64)	Non-NAFLD (*n* = 85)	*p* value
Socio-demographics			
Age (years)	65 (58–72)	67 (58–74)	0.474
Female sex, *n* (%)	34 (53.1)	41 (48.2)	0.555
Married, *n* (%)	58 (90.6)	67 (78.8)	0.052
Education level *n* (%)			0.175
Primary school or below	40 (62.5)	62 (72.9)	
High school or above	24 (37.5)	23 (27.1)	
Medical histories			
Hypertension, *n* (%)	50 (78.1)	50 (58.8)	0.013
Diabetes, *n* (%)	32 (50.0)	25 (29.4)	0.010
Hyperlipidemia *n* (%)	37 (57.8)	37 (43.5)	0.084
Risk factors			
Smoking, *n* (%)	12 (18.8)	22 (25.9)	0.304
BMI (kg/m^2^)	24.44 (24.03–27.50)	23.93 (21.70–24.03)	<0.001
SBP (mmHg)	152.19 ± 20.73	150.41 ± 20.83	0.607
DBP (mmHg)	85.09 ± 12.07	85.75 ± 12.63	0.748
MAP (mmHg)	107.46 ± 13.15	107.31 ± 12.88	0.944
Laboratory indexs			
CRP (mg/L)	3.30 (2.99–7.04)	3.30 (2.98–3.95)	0.115
Hs-CRP (mg/L)	2.57 (0.98–5.16)	1.10 (0.55–3.01)	0.001
Folic acid (ng/mL)	9.17 (7.07–11.97)	9.83 (6.24–13.57)	0.898
Vitamin B12 (pg/mL)	360 (243–479)	315 (218–427)	0.163
D-dimer (mg/L)	0.37 (0.25–0.58)	0.33 (0.24–0.58)	0.617
Troponin (ng/mL)	0.012 (0.012–0.017)	0.012 (0.012–0.017)	0.922
FBG (mmol/L)	6.37 (5.00–8.30)	4.97 (4.55–6.09)	<0.001
HbA1c (%)	6.55 (5.67–7.62)	5.79 (5.40–6.60)	0.002
ALT (U/L)	18 (13–26)	15 (11–18)	<0.001
AST (U/L)	18 (16–25)	18 (15–21)	0.263
Creatinine (μmol/L)	60.5 (48.6–75.4)	61.2 (51.8–72.9)	0.866
Uric acid (μmol/L)	308 (251–370)	278 (228–330)	0.030
Hcy (μmol/L)	11.3 (9.1–13.2)	10.6 (8.2–12.6)	0.203
TG (mmol/L)	1.83 (1.33–2.60)	1.34 (0.99–1.80)	<0.001
TC (mmol/L)	4.51 ± 1.10	4.47 ± 0.99	0.942
HDL (mmol/L)	0.95 (0.77–1.14)	1.04 (0.88–1.28)	0.009
LDL (mmol/L)	2.63 (2.12–3.39)	2.80 (2.19–3.46)	0.452
FT3 (pg/mL)	2.97 (2.65–3.15)	2.97 (2.77–3.26)	0.325
FT4 (ng/dL)	1.16 (1.06–1.29)	1.18 (1.05–1.30)	0.855
TSH (μIU/mL)	2.042 (1.253–2.627)	2.102 (1.235–2.708)	0.899
At admission			
HAMA score	9 (5–12)	10 (6–13)	0.556
HAMD score	7 (3–10)	7 (4–13)	0.369
PSQI score	3 (2–6)	3 (2–7)	0.393
MMSE score	24 (21–27)	23 (20–26)	0.219
NIHSS score	2 (1–4)	2 (1–4)	0.989
At follow-up			
FD, *n* (%)	29 (45.3)	20 (23.5)	0.005

SBP, systolic blood pressure; DBP, diastolic blood pressure; MAP, mean arterial pressure; FT3, free triiodothyronine; FT4, free thyroid hormone; TSH, thyroid stimulating hormone.

**Table 2:** Multivariate logistic regression analysis.

Parameter	*p* value	OR	95% CI
NAFLD	0.043	2.359	1.027–5.420
Female	0.014	2.910	1.244–6.806
NIHSS score	< 0.001	1.461	1.218–1.752

### 3.2 The comparison of gut microbiota diversity between NAFLD and non-NAFLD groups

**Figure 1:**
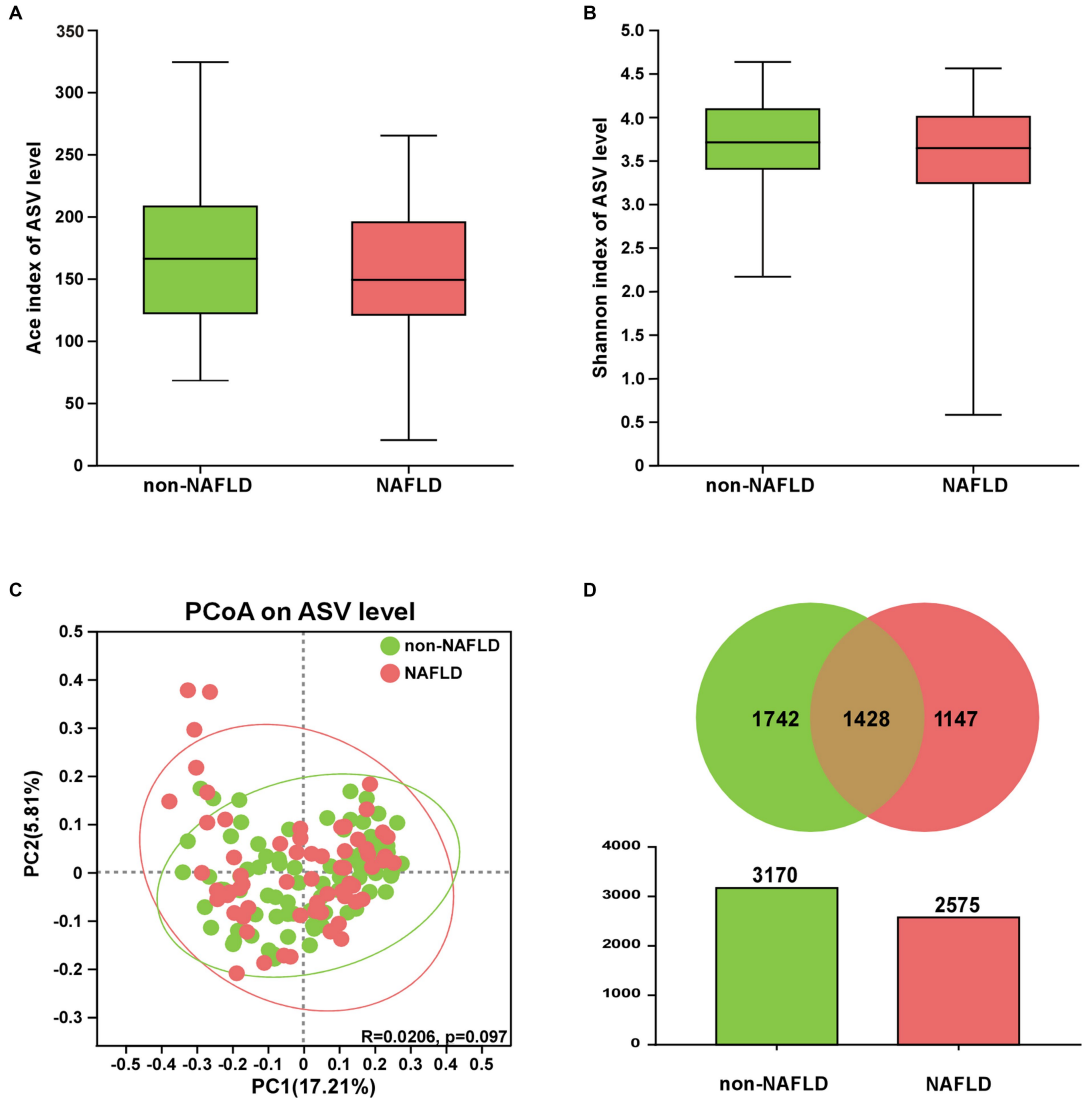
The comparison of gut microbiota diversity between NAFLD and non-NAFLD groups. **(A)** ACE index and **(B)** Shannon index. **(C)** PCoA of gut microbiota based on the unweighted-unifrac (PC1 = 17.21%, PC2 = 5.81%). **(D)** The number of ASVs was displayed via Venn and Colum diagrams.

As shown in Figures 1A,B, there were no significant differences in ACE richness and Shannon diversity between NAFLD and non-NAFLD groups. As shown in Figure 1C, clustering of the two groups were exhibited through PCoA scatterplot representing beta diversity of gut microbiota. As shown in Figure 1D, a total of 4,317 differential ASVs were detected in the two groups. Furthermore, the ASV number of non-NAFLD group (3,170 ASVs) was richer than NAFLD group (2,575 ASVs).

### 3.3 The comparison of gut microbiota composition between NAFLD and non-NAFLD groups

**Figure 2:**
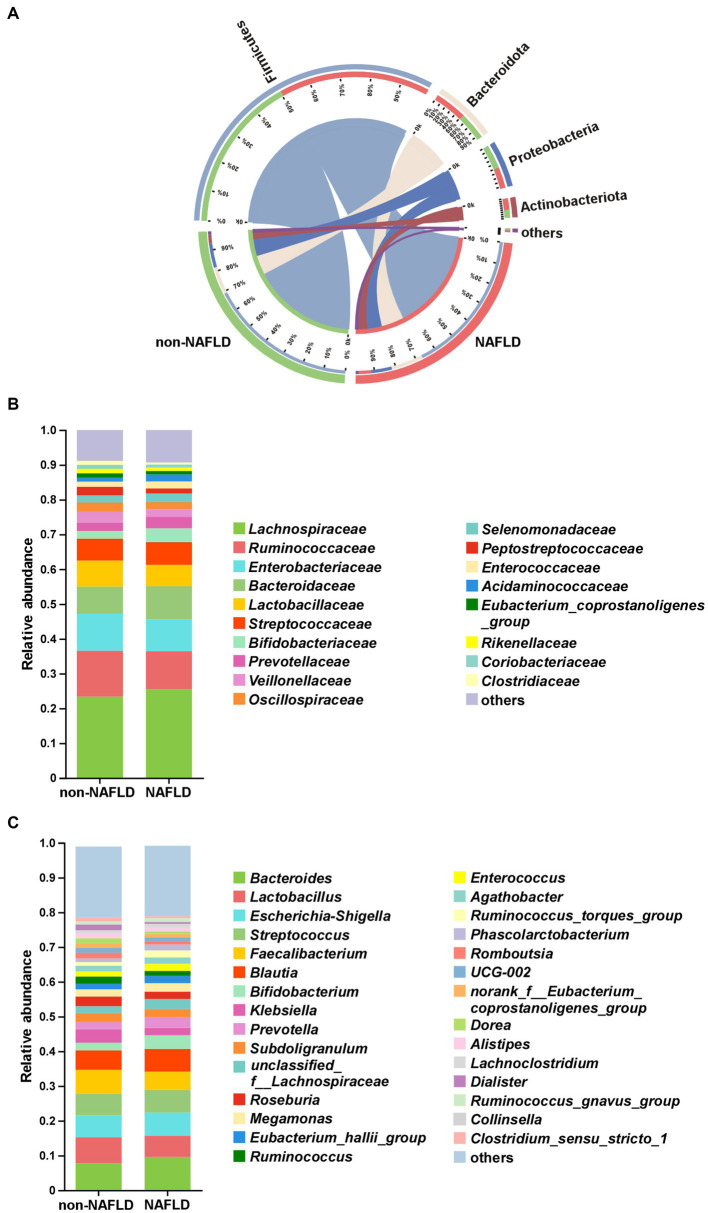
The comparison of gut microbiota composition between non-NAFLD and NAFLD groups. **(A)** Distribution of the microbial community at the phylum level. Average relative abundances of microbial community composition were shown by bar plots for the family level **(B)** and genus level **(C)**.

As shown in Figure 2A, Firmicutes, Bacteroidota, Proteobacteria and Actinobacteriota were the main components of gut microbiota at the phylum level in both groups. At the family level, the top 5 bacteria in relative abundance were *Lachnospiraceae*, *Ruminococcaceae*, *Enterobacteriaceae*, *Bacteroidaceae*, *Lactobacillaceae* in both groups (Figure 2B). At the genus level, the NAFLD group showed increased relative abundance of *Bacteroides* but decreased relative abundance of *Lactobacillus* (Figure 2C).

### 3.4 Characteristic of gut microbiota in AIS patients with NAFLD

**Figure 3:**
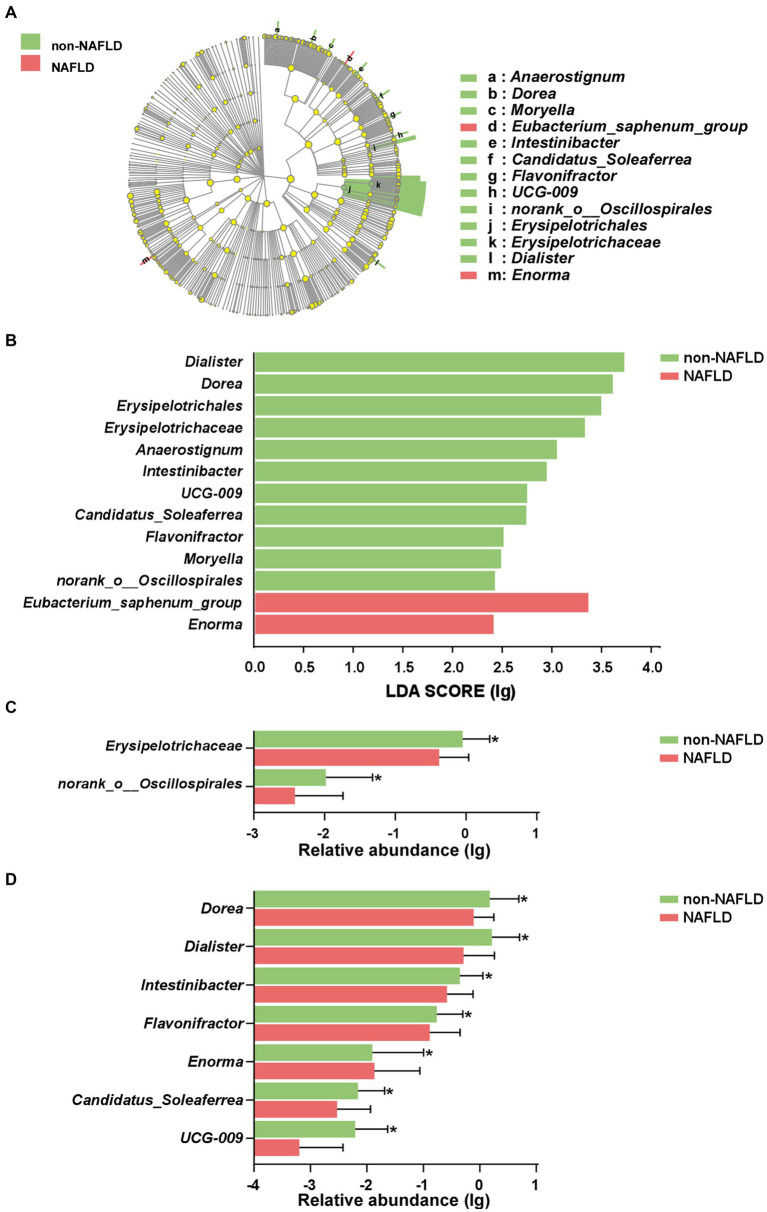
Characteristic of gut microbiota in AIS patients with NAFLD. **(A)** Cladograms were representing the LEfSe. The center dot of cladogram represented the root of the tree, and each ring represented the next lower taxonomic level (phylum to genus). **(B)** LDA histogram of gut microbiota. Taxa with an LDA score > 2 were listed. The column diagram of significant different taxa at the family **(C)** and genus **(D)** levels. ^*^p < 0.05.

As shown in Figures 3A,B, the cladogram and LDA discriminant histogram displayed 13 important characteristic microbiota taxa with detailed annotation between the two groups. The family taxonomic proportion of *Erysipelotrichaceae* and *norank_o__Oscillospirales* were richer in AIS patients without NAFLD compared to the NAFLD group (Figure 3C). Among the top 7 genera in relative abundance with significant differences, the abundance *Dorea*, *Dialister*, *Intestinibacter*, *Flavonifractor*, *Candidatus_Soleaferrea* and *UCG-009* were significantly lower in NAFLD group compared with non-NAFLD group, but *Enorma* was significantly higher in relative abundance in NAFLD group compared with non-NAFLD group (Figure 3C).

### 3.5 The correlation between gut microbiota and clinical parameters

**Figure 4:**
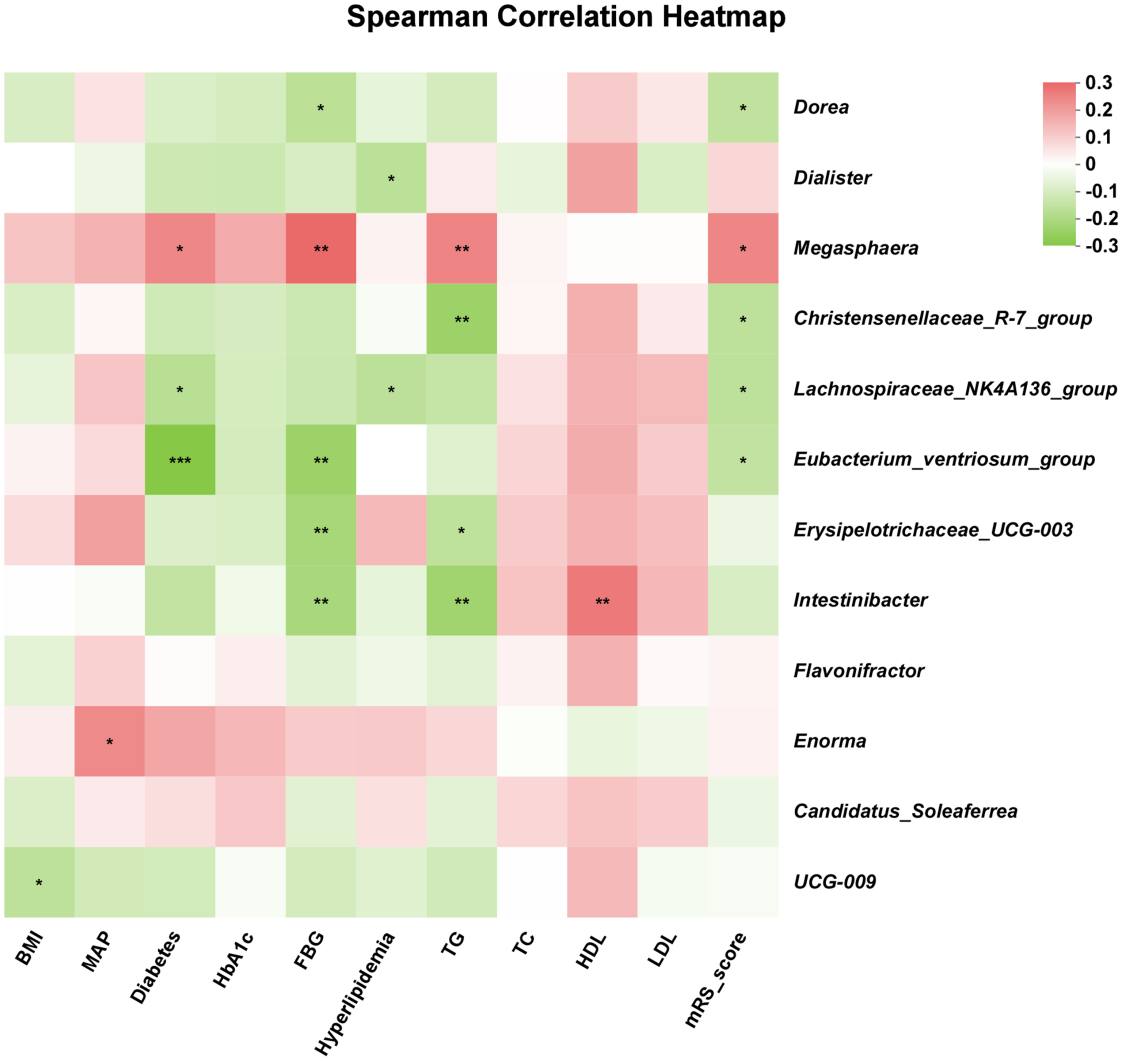
The correlation between gut microbiota and clinical parameters. Red color indicated positive correlation and green color indicated opposite. The lighter red or green indicated weaker correlation values. ^*^p < 0.05; ^**^p < 0.01; ^***^p < 0.001.

As shown in Figure 4, the relationship between specific microbiota and clinical parameters was presented. The abundance of *Dorea* was negatively correlated with mRS score and FBG level. In addition, the abundance of *Enorma* was positively correlated with MAP, and the abundance of *UCG-009* was negatively correlated with BMI. Moreover, TG level was positively associated with the abundance of *Megasphaera* but was negatively associated with the abundance of *Christensenellaceae_R-7_group*, *Erysipelotrichaceae_UCG-003*, and *Intestinibacter*, and HDL level was positively associated with the abundance of *Intestinibacter*. The abundance of *Eubacterium_ventriosum_group*, *Erysipelotrichaceae_UCG-003* and *Intestinibacter* were negatively correlated with the FBG level, but the abundance of *Megasphaera* was positively correlated with the FBG level.

### 3.6 The prediction for prognosis of patients in AIS with NAFLD

**Figure 5:**
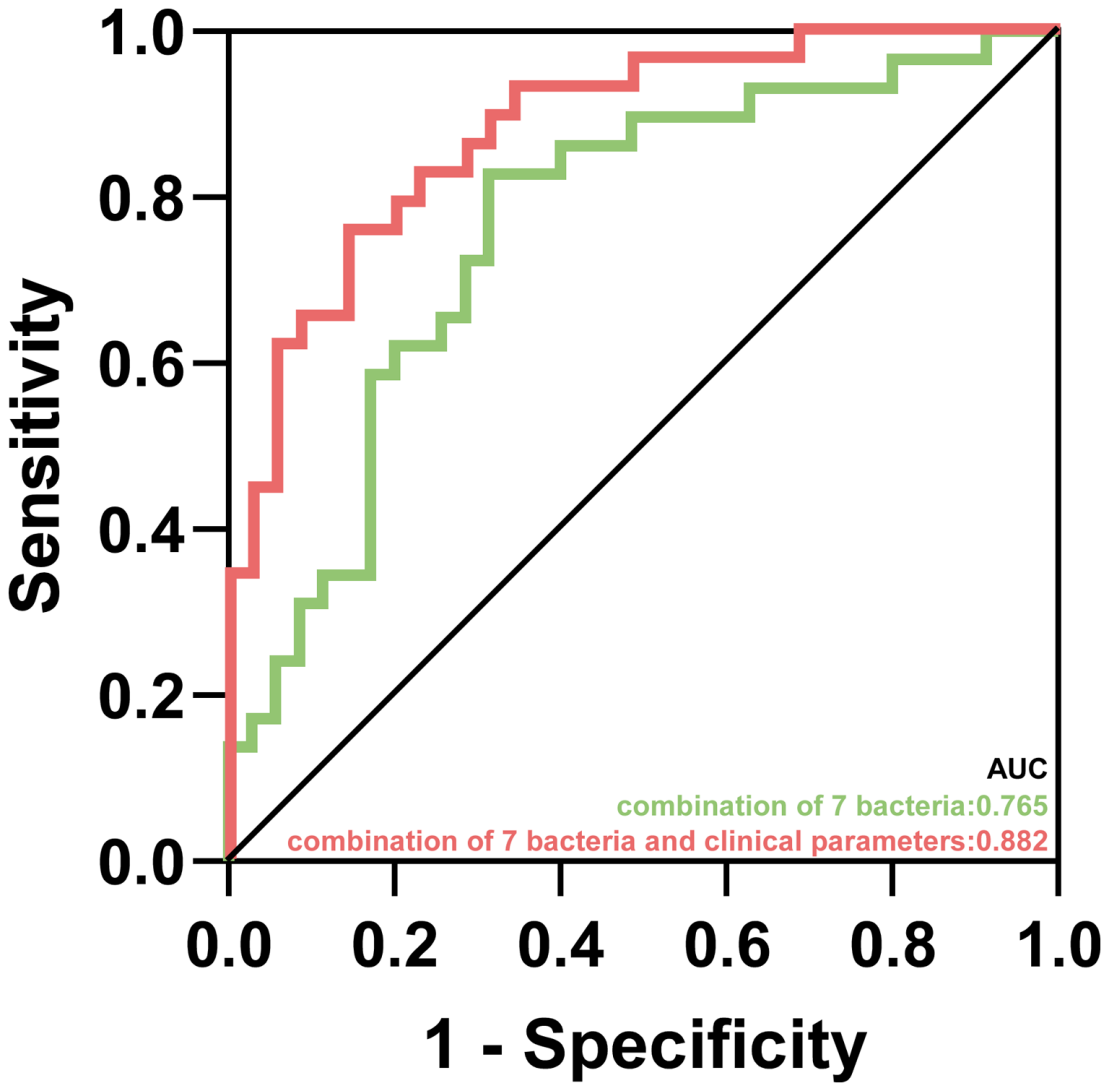
The prediction for prognosis of patients in AIS with NAFLD. Predictive capability of characteristic gut microbiota in AIS patients with NAFLD group. Green line represented the combination of 7 characteristic bacteria. Red line represented the combination of 7 characteristic bacteria and clinical parameters.

As showed in Figure 5, we conducted two predictive models based on 7 characteristic bacteria in AIS with NAFLD and clinical independent risk factors (NIHSS score and female) from multivariable logistic regression. The AUC value of combination of 7 characteristic bacteria yielded to 0.765 (*p* < 0.001, 95% CI: 0.646–0.883). Moreover, the predictive model combined 7 genera and 2 clinical independent risk factors could distinguish FD from FI in AIS patients with NAFLD (*p* < 0.001, AUC = 0.882, 95% CI: 0.801–0.963).

## 4 Discussion

In this study, we revealed the features of gut microbiota in AIS patients with NAFLD, and then demonstrated the close relationship between characteristic bacteria and clinical parameters, as well as the ability of characteristic bacteria as biomarkers to effectively distinguish FD patients in AIS with NAFLD.

A cross-sectional study shows that cerebrovascular diseases are the most common among NAFLD patients (Terai et al., 2021). In this study, the existence of NAFLD in AIS patients was an independent risk factor for FD at 3 months post-stroke. A meta-analysis showed that the NAFLD significantly contributed to a higher incidence of AIS and it was further confirmed in the stratified analysis by ethnicity and cerebrovascular disease classification (Hu et al., 2018). Furthermore, an important finding of a previous study showed that the mRS score of AIS patients with NAFLD were higher than the AIS without NAFLD at discharge (Abdeldyem et al., 2017).

Emerging studies have shown close relationship between gut microbiota and neurological diseases. A cross-sectional study showed microbiota composition disorder in ischemic stroke patients compared with healthy subjects (Li et al., 2019). Moreover, gut microbiota dysregulation contributed to metabolic disorders including NAFLD (Luo et al., 2023), and gut microbiota were potentially involved in the pathogenesis of AIS and NAFLD. In this study, the abundance of *Dorea* was significantly decreased in AIS patients with NAFLD, was negatively corelated with mRS score, which indicated that the reduced abundance of *Dorea* might be a risk factor for adverse outcome. *Dorea* is a member of *Lachnospiraceae* family. The present observation was consistent with a prior study, the abundance of *Dorea* decreased in AIS group with worse outcome compared to control group, and further revealed the improvement of the neurological impairment was associated with *Dorea* (Guo et al., 2022). In addition, it has been reported that the increase in abundance of *Dorea* can be considered a crucial protective factor, and the abundance of *Dorea* decreased in NAFLD patients with BMI < 30 kg/m^2^ (Juárez-Fernández et al., 2023). In addition, the abundance of *Dorea* in NAFLD patients increased after probiotic treatment, and the abundance of *Dorea* was positively correlated with the improvement of NAFLD (Juárez-Fernández et al., 2023). In this study, LEfSe analysis had shown that *Intestinibacter* was significantly enriched in non-NAFLD group, and *Intestinibacter* was negatively related with the FBG and TG. In line with these results, a reduced abundance of *Intestinibacter* was observed in Parkinson’s disease (Kenna et al., 2021). In addition, *Intestinibacter* and *Dorea* were negatively correlated with PSQI score in patients with major depressive disorder (Zhang et al., 2021). *Intestinibacter bartlettii*, a species of *Intestinibacter* genera, had been previously reported lower in overweight and obese children who were at higher risk of developing metabolic disorders (Murga-Garrido et al., 2023). Of note, *Dorea* and *Intestinibacter* were generally considered as beneficial bacteria producing SCFAs, which have anti-inflammatory properties and ameliorate endothelial dysfunction by inhibiting the production of pro-inflammatory cytokines (Li M. et al., 2018; Chen et al., 2021; Yi et al., 2021). The abundance of SCFAs-producing bacteria decreased in the population at a high risk of stroke (Zeng et al., 2019). The levels of SCFAs were negatively correlated with neurological impairment and infarction volume in cerebral ischemic rat models (Chen et al., 2019). A cross-sectional study observed a negative relationship between the circulating level of SCFAs and the severity of NAFLD (Tsai et al., 2023). SCFAs, especially acetic acid, could modulate the hepatic lipids metabolism to alleviate the hepatic steatosis in NAFLD mice (Hong et al., 2021). These finding suggested that gut microbiota producing SCFAs might be protective factors for the development and progression of AIS patients with NAFLD.

In addition, chronic systemic inflammation was a vital feature and crucially participated in the pathophysiology of the development and prognosis of NAFLD and AIS (Gehrke and Schattenberg, 2020; Endres et al., 2022). In NAFLD patients, a decrease in bacteria producing SCFAs and an increase of gut barrier permeability were observed (Miele et al., 2009). SCFAs reduction might have a detrimental role in the whole setting of systemic inflammation (Juanola et al., 2019). NAFLD associated with dysbiosis could potentiate a systemic pro-inflammatory environment and then unfavorably influence the outcome of AIS. Previous studies reported that excessive inflammatory responses were harmful to neuronal function (Russo and McGavern, 2016). Mice transplanted with cirrhosis patients gut microbiota rather than healthy human had the higher levels of neuroinflammation and glial activation (Liu et al., 2020). The balance of interaction among the liver, gut and brain, might act as an important function in improving systematic inflammatory responses. The gut microbiota dysbiosis and upregulation of inflammatory response play a crucial role in adverse outcomes in AIS patients with NAFLD. These results suggested that alternation of gut microbiota participated in the process of NAFLD influenced the prognosis of AIS patients, and some characteristic bacteria can not only provide candidate biomarkers for disease diagnosis, but also for subsequent mechanism research. However, it is difficult to prove the causal relationship between characteristic bacterial and the occurrence and development of diseases. In future research, it is necessary to better reveal the relationship between gut microbiota and AIS patients with NAFLD.

This study also has several limitations. Although probable confounding factors such as socio-demographic indicators had been eliminated, stress and drug intake could affect gut microbiota of participants. In addition, the assessment of functional outcome based on mRS in the follow-up needs to unify more factors that may also affect functional prognosis of patients, such as rehabilitation training. This study was a time point measurement of gut microbiota, and multiple time points should be taken to reveal the dynamic changes of gut microbiota in AIS patients with NAFLD. Moreover, the metabolic and inflammatory changes of gut microbiota should be further studied to clarify the possible underlying mechanisms.

In conclusion, these findings elucidated the microbial characteristics in AIS patients with NAFLD, further revealed the predictive capability of characteristic microbiota for adverse outcomes, and provided novel potential predictive tools for adverse outcome in AIS patients with NAFLD.

## Data availability statement

The datasets presented in this study can be found in online repositories. The names of the repository/repositories and accession number (s) can be found at: https://www.ncbi.nlm.nih.gov/, PRJNA1030888.

## Ethics statement

The studies involving humans were approved by the Medical Ethics Committee of the Second Affiliated Hospital of Wenzhou Medical University. The studies were conducted in accordance with the local legislation and institutional requirements. Written informed consent for participation in this study was provided by the participants’ legal guardians/next of kin.

## Author contributions

GY: Writing – original draft, Data curation, Writing – review & editing, Conceptualization, Software, Visualization, Formal analysis, Methodology, Validation. QC: Writing – original draft, Writing – review & editing, Data curation, Software, Methodology, Validation, Visualization. JC: Writing – review & editing, Investigation. XL: Writing – review & editing, Investigation. HX: Writing – review & editing, Software, Visualization. YZ: Writing – review & editing, Data curation, Investigation. JL: Writing – review & editing, Funding acquisition, Conceptualization, Supervision. JS: Writing – review & editing, Funding acquisition, Conceptualization, Supervision. SC: Writing – review & editing, Funding acquisition.
